# Mutation in the *pssZ* Gene Negatively Impacts Exopolysaccharide Synthesis, Surface Properties, and Symbiosis of *Rhizobium leguminosarum* bv. *trifolii* with Clover

**DOI:** 10.3390/genes9070369

**Published:** 2018-07-23

**Authors:** Paulina Lipa, José-María Vinardell, Joanna Kopcińska, Agnieszka Zdybicka-Barabas, Monika Janczarek

**Affiliations:** 1 Department of Genetics and Microbiology, Institute of Microbiology and Biotechnology, Faculty of Biology and Biotechnology, Maria Curie-Skłodowska University, Akademicka 19 St., 20-033 Lublin, Poland; paulina.lipa56@gmail.com; 2 Department of Microbiology, Faculty of Biology, University of Sevilla, Avda. Reina Mercedes 6, 41012 Sevilla, Spain; jvinar@us.es; 3 Department of Botany, Faculty of Agriculture and Biology, Warsaw University of Life Sciences, Nowoursynowska 166 St., 02-787 Warsaw, Poland; j.kopcinska@wp.pl; 4 Department of Immunobiology, Institute of Biology and Biochemistry, Faculty of Biology and Biotechnology, Maria Curie-Skłodowska University, Akademicka 19 St., 20-033 Lublin, Poland; barabas@poczta.umcs.lublin.pl

**Keywords:** *Rhizobium leguminosarum*, *pssZ*, serine/threonine protein phosphatase, exopolysaccharide synthesis, cell-surface properties, symbiosis, clover

## Abstract

*Rhizobium leguminosarum* bv. *trifolii* is a soil bacterium capable of establishing a nitrogen-fixing symbiosis with clover plants (*Trifolium* spp.). This bacterium secretes large amounts of acidic exopolysaccharide (EPS), which plays an essential role in the symbiotic interaction with the host plant. This polymer is biosynthesized by a multi-enzymatic complex located in the bacterial inner membrane, whose components are encoded by a large chromosomal gene cluster, called Pss-I. In this study, we characterize *R. leguminosarum* bv. *trifolii* strain Rt297 that harbors a Tn*5* transposon insertion located in the *pssZ* gene from the Pss-I region. This gene codes for a protein that shares high identity with bacterial serine/threonine protein phosphatases. We demonstrated that the *pssZ* mutation causes pleiotropic effects in rhizobial cells. Strain Rt297 exhibited several physiological and symbiotic defects, such as lack of EPS production, reduced growth kinetics and motility, altered cell-surface properties, and failure to infect the host plant. These data indicate that the protein encoded by the *pssZ* gene is indispensable for EPS synthesis, but also required for proper functioning of *R. leguminosarum* bv. *trifolii* cells.

## 1. Introduction

*Rhizobium leguminosarum* bv. *trifolii* is a Gram-negative bacterium that exists as a free-living organism in the soil or establishes nitrogen-fixing symbiosis with clover plants (*Trifolium* spp.). This microorganism belongs to a large and diverse group of soil bacteria, collectively called rhizobia, which possess the ability to induce nodules on roots and stems of legumes [1,2]. Within nodules, new plant organs ensuring a special ecological niche, rhizobia reduce dinitrogen to ammonia, which is then used by the host plant. The nitrogen-fixing symbiosis is a highly specific and complex process, which involves many signals of plant and bacterial origin; among such signals, flavonoids secreted by legume roots, and rhizobial lipochitooligosaccharides and exopolysaccharides (EPS) play crucial roles [1,3].

Recent findings indicate that the function of EPS in the legume–rhizobium symbiosis is more complex than initially anticipated and depends largely on the host plant. In general, this polysaccharide is required for effective symbiosis of bacteria with a great majority of legumes, which form indeterminate-type nodules (e.g., clover, vetch, pea, and alfalfa) [1,4,5]. The significance of EPS in the symbiosis with this type of legumes is confirmed by the symbiotic phenotype of rhizobial strains defective in EPS production (e.g., *R. leguminosarum* bvs. *trifolii* and *viciae* and *Sinorhizobium meliloti*). These strains are only able to induce the formation of small, partially infected or even empty, nodule-like structures on the compatible host plants that are ineffective in nitrogen fixation [6,7,8,9]. However, some exceptions were found, e.g., *Sinorhizobium fredii* strain HH103, whose EPS was shown to not be required for nodulation of *Glycyrrhiza uralensis*, a host plant that also forms indeterminate-type nodules [10,11]. On the other hand, although EPS can be dispensable for the symbiosis with legumes that form determinate nodules (such as *S. fredii*-soybean symbiosis) [12], it was recently shown that *Mesorhizobium loti* EPS is an important signal for symbiotic interactions with the hosts *Lotus corniculatus* and *Lotus japonicus*, which form determinate-type nodules [13,14,15]. In fact, it has been demonstrated in this symbiosis that the recognition of the appropriate EPS by a legume receptor is required for proper infection of host plant roots [15]. Apart from being a symbiotic signal required for the initiation and elongation of infection threads (ITs; special tubular structures via which rhizobia colonize root nodules), EPS provides protection against host plant defense reactions. Moreover, this polymer plays several other functions in free-living rhizobia, such as nutrient gathering, biofilm formation, and protection against desiccation and other stress factors, ensuring adaptation of these bacteria to changing environmental conditions [4,16,17].

The chemical structure of EPS synthesized by *R. leguminosarum* has been determined in detail. This polymer is composed of octasaccharide repeating subunits that contain d-glucose, d-glucuronic acid, and d-galactose residues in a molar ratio 5:2:1, and are additionally substituted with *O*-acetyl and pyruvyl groups [18,19,20,21,22]. EPS is synthesized in high-molecular weight and low-molecular weight forms. However, data about the genetic control of EPS production in *R. leguminosarum* are only fragmentary. So far, only the function of a few proteins involved in the synthesis and export of EPS have been experimentally confirmed. This polysaccharide is biosynthesized by a large multi-enzymatic complex located in the bacterial inner membrane (IM). PssA is involved in the initiation of the EPS subunit assembly. This enzyme transfers glucose-1-phosphate from UDP-glucose to a lipid undecaprenylphosphate (und-PP) carrier anchored in the bacterial IM [23]. PssDE [glucuronosyl-(β1,4)-glucosyltransferase], PssC [glucuronosyl-(β1,4)-glucuronosyltransferase], and PssS [glucosyl-(α1,4)-glucuronosyltransferase] are engaged in the subsequent three steps of the EPS unit assembly. These proteins are encoded by genes located in a large chromosomal Pss-I cluster [24,25,26]. Mutations in the *pssA*, *pssD*, *pssE*, or *pssS* genes totally abolish EPS synthesis [6,7,9,25,26]. Moreover, the protein encoded by *pssJ* is probably involved in the last step of the subunit synthesis, since the *exo*344::Tn*5* strain that harbors a mutation in this gene only produces residual amounts of structurally altered EPS, whose units were lacking the terminal d-galactose [21,22]. However, the enzymes involved in the remaining steps of EPS synthesis have not yet been identified. Based on sequence similarities between Pss proteins and enzymes available in the protein databases [PDB, CAZy] and on the phenotypes of several *pss* mutants, Ivashina and Ksenzenko [24] postulated that the subsequent steps of EPS subunit assembly might be carried out by PssF, PssI/PssG, and PssH/PssI glycosyltransferases, encoded by genes located in the Pss-I cluster. Moreover, the PssR, PssM, and PssK proteins are most probably involved in non-sugar modifications of EPS. Among them, PssM, which exhibits ketal pyruvate transferase activity, adds pyruvyl groups to the subterminal sugar residue in the repeating units [27], whereas PssR and PssK are responsible for the addition of *O*-acetyl and pyruvyl groups to the second and eight sugar residues, respectively [24]. Furthermore, several genes involved in EPS polymerization and secretion have been characterized (*pssTNOP, pssL*, and *pssP2*); all of these, except for *pssP2*, are also located in the Pss-I region [28,29,30]. It is well known that rhizobial EPS is synthesized by a Wzx/Wzy-dependent mechanism in which the subunits are assembled in the cytoplasmic leaflet of the IM, and then transported to the periplasmic leaflet of the IM for polymerization and subsequent secretion [31,32]. This mechanism involves two key proteins, Wzx (flippase) and Wzy (polymerase). In the case of *R. leguminosarum*, the Wzx flippase and Wzy polysaccharide polymerase are encoded by the *pssL* and *pssT* genes, respectively. In addition, polysaccharide co-polymerase (PCP, also classified as a membrane periplasmic auxiliary protein, MPA), encoded by the *pssP* gene, determines the length of EPS chains [4,29,30].

Recently, PssP and other members of the PCP group involved in bacterial EPS synthesis were classified as bacterial tyrosine kinases with autophosphorylating kinase activity [33]. This activity has been shown to be essential for the oligomerization of these proteins, and, consequently, for EPS production and regulation of the polymer chain length [34]. Despite these findings, the mechanism determining the strain-specific chain length of EPS is still not well understood. Tocilj et al. [35] proposed a model dependent on the stoichiometry of the protein complex comprising PCP. Moreover, oligomers of Wza-type proteins (lipoproteins), forming a channel in the outer membrane (OM), and interacting with the Wzx and Wzy proteins, are engaged in transporting the EPS out of the cells through the OM. In *R. leguminosarum*, this Wza-type protein is PssN [4,29,36]. It is thought that the mechanisms of biosynthesis and translocation of polysaccharides outside the cell must be temporally and spatially coordinated. This could be achieved by interactions of different components of this multi-protein complex. In fact, most probably, glycosyltransferases interact with the flippase and the co-polymerase, and regulation of the chain length could take place at this stage [37]. The glucosyltransferase PssA, which initiates the process of EPS synthesis and is a key element of this enzymatic machinery, is proposed as a highly probable site for these interactions. This protein contains several serine and threonine residues, which might be potential sites for phosphorylation. Moreover, other proteins influencing the enzymatic activity and/or protein-protein interactions might additionally modulate the function of this EPS-synthesizing machinery.

In the current study, we have characterized a *R. leguminosarum* bv. *trifolii* strain harboring a mutation in the *pssZ* gene, which is located in the Pss-I region. We demonstrate that this gene plays an essential role in EPS synthesis, and affects different cell-surface properties and symbiosis of *R. leguminosarum* bv. *trifolii* with clover.

## 2. Materials and Methods

### 2.1. Bacterial Strains, Plasmids, and Growth Conditions

Bacterial strains, plasmids and primers used in this study are listed in Table 1.

*Rhizobium leguminosarum* strains were grown in 79CA medium supplemented with 1% glycerol at 28 °C with agitation (160 rpm) [45], whereas *Escherichia coli* strains were cultured in Luria-Bertani (LB) medium at 37 °C [40]. When required, the media were supplemented with the appropriate antibiotics used at the following final concentrations: kanamycin, 40 μg mL^−1^, ampicillin, 100 μg mL^−1^, and nalidixic acid, 40 μg mL^−1^. To compare growth kinetics of the studied strains, bacteria were cultured over 72 h and after 0, 24, 48, and 72 h, the optical density (OD_600_) of these cultures was measured. Then, 100-μL aliquots were taken and their serial dilutions placed onto agar plates, incubated for 4 d, and the appearing colonies (colony-forming units, CFU) were counted. The experiment was repeated twice with three biological replicates for each strain tested.

### 2.2. DNA Methods and Sequence Analysis

Standard molecular techniques, such as genomic and plasmid DNA isolation, restriction enzyme digestion, cloning, hybridization, and transformation were performed according to [40]. For PCR reactions, Ready-to-use RED-*Taq* DNA polymerase mix (Sigma-Aldrich, St. Louis, MO, USA) and primers listed in Table 1 were used. Sequencing was performed using the BigDye terminator cycle sequencing kit (Applied Biosystems, Foster City, CA, USA) and the ABI Prism 310 apparatus. Database searches were done with the FASTA and BLAST programs at the National Center for Biotechnology Information (Bethesda, MD, USA) and the European Bioinformatic Insitute (Hinxton, UK) [46,47]. Promoter prediction in the *pssZ* regulatory region was done using the BDGP (Berkeley Drosophila Genome Project) Neural Network Promoter Prediction [48] (Berkley, CA, USA), as well as using the Malign ver. 3.0 and Fuzznuc ver. 2.10 programs [49,50] and the *S. meliloti* CTTGAC-N_17-18_-CTATAT and *E. coli* TTGACA-N_17-18_-TATAAT promoter consensus sequences as a query [51]. Amino acid sequence analyses were performed using the BLASTP program [47].

### 2.3. Isolation of a Rhizobium leguminosarum pssZ Mutant

Strain Rt297 was obtained as a result of a random mutagenesis of the wild-type strain Rt24.2, which was performed using *E. coli* S17-1 containing the mTn*5*SS*gusA*40 transposon with a promoterless *gusA* gene as a donor [42]. Localization of the transposon in the Rt297 genome was determined by hybridization with a *gusA* probe, and PCR analyses with the use of primers complementary to different regions of the Pss-I cluster and the 5′-end of *gusA*. PCR products (from 1.5- to 1.9-kb long) were obtained by using primer pairs: gusR1/J44-Rw4, gusR1/J44-Rw5, gusR2/J44-Rw4, gusR2/J44-Rw5, and gusR2/Eco-Rw1. Based on sequencing analysis of these amplicons, the mTn*5*SS*gusA*40 insertion was located within *pssZ* from the Pss-I region.

### 2.4. Construction of Plasmid pPL1 for Complementation of the pssZ Mutation

To construct a plasmid containing the entire *pssZ* gene including its upstream region, the pBBR1MCS-2 vector and a 1.8-kb long amplicon obtained in the PCR reaction with primers Sal-Rw2/Xba-Fw3 (Table 1) were used. The PCR product was digested with XbaI and SalI enzymes and ligated to the pBBR1MCS-2 vector digested with the same endonucleases. The resulting plasmid, pPL1, was verified by sequencing. Next, pPL1 was introduced into *E. coli* S17-1 by transformation and subsequently into Rt297 by biparental conjugation according to [25].

### 2.5. β-glucuronidase Assay

Assays for β-glucuronidase activity were carried out according to the protocol described by Miller [52] using 24-h bacterial cultures and p-Nitrophenyl-β-d-glucuronide as a substrate (Sigma-Aldrich, St. Louis, MO, USA) [53]. The reported values are given in Miller units and are averages of two independent experiments with three replicates for each strain tested.

### 2.6. Isolation and Quantification of Exopolysaccharide

Cultures of rhizobial strains (5 mL) were grown in 79CA for 48 h. Then, the cultures were centrifuged (20 min, 12,000× *g*) and EPS was precipitated from the obtained supernatants using 4 vol. (for high-molecular weight, HMW EPS) or 10 vol. of 96% cold ethanol (for low-molecular weight, LMW EPS), accordingly, collected by centrifugation, dissolved in deionized water and analyzed for carbohydrates according to [54]. The total sugar content was calculated as glucose equivalents. The experiment was repeated twice with three replicates for individual strain.

### 2.7. Determination of Lipopolysaccharide Profiles

Lipopolysaccharide (LPS) profiles of the tested strains were determined as reported previously [26]. Briefly, 2-d bacterial cultures were centrifuged and pellets obtained were washed twice with 0.9% NaCl to remove EPS, and used to determine LPS profiles according to [55]. Samples were separated in 12.5% Tricine SDS polyacrylamide gel electrophoresis (SDS-PAGE), and LPS in gels was visualized by silver staining.

### 2.8. Cell Hydrophobicity Assay

The hydrophobicity of rhizobial strains was determined using a two-phase method and dodecane (Sigma-Aldrich, St. Louis, MO, USA) according to [56]. For this assay, bacterial pellets obtained from 24 h cultures and resuspended in PUM buffer were used. The degree of hydrophobicity was calculated as follows: % hydrophobicity = 100 − 100(OD_a_/OD_1_), where (OD_1_) is the optical density at 405 nm of bacterial suspensions before adding dodecane and OD_a_ is the optical density of these cultures after a 15 min incubation with dodecane. The experiment was performed twice with three replicates for each strain analyzed.

### 2.9. Aggreagation Assay

The aggregation of rhizobial cells was determined according to the method described by Sorroche et al. [57] with a slight modification [58]. For this experiment, 24 h cultures of similar optical density (~OD_600_ = 0.6) were left for 24 h without agitation at room temperature. Next, 300 μL of the upper phase of these samples were collected and their OD_600_ was measured (OD_A2_) in a microplate reader (Biochrom Asys UVM 340). The remaining samples were extensively vortexed and their OD_600_ was measured (OD_A1_). The degree of aggregation was calculated as follows: % aggregation = 100 -* 100(OD_A2_/OD_A1_). The assay was repeated twice with three replicates for each strain tested.

### 2.10. Determination of Cell Motility

For this purpose, 5 μL aliquots of bacterial suspensions of an optical density OD_600_ = 0.2 prepared in sterile water were stabbed into 0.3% 79CA agar (swimming) or placed on the surface of 0.7% 79CA agar (surface motility). Then, the plates were incubated at 25 °C for 15 days, and the migration distance from the site of bacterial addition was measured after 3, 6, 9, and 15 days. The assay was repeated twice with three replicates for each strain examined.

### 2.11. Determination of Rhizobial Sensitivity to Stress Factors

To compare tolerance of rhizobial strains to several stress factors [SDS, sodium deoxycholate (DOC), and ethanol], the minimal inhibitory concentration of the individual component was determined. For this purpose, 10 μL aliquots of bacterial suspensions of OD_600_ = 0.2 prepared in sterile water were placed on 79CA agar plates containing different concentrations of the tested compounds (SDS: 0.05–1% *w*/*v*, DOC: 0.05–1% *w*/*v*, ethanol: 0.05–6% *v*/*v*). The bacterial growth on the individual media was determined after 48 h. The experiment was repeated three times with three replicates for each strain and condition tested.

### 2.12. Biofilm Production Assay

Biofilm formation assays were performed according to a method described by Rinaudi and Gonzalez [59]. Briefly, 24 h bacterial cultures were diluted to OD_600_ = 0.4 and 100 μL aliquots were added to polystyrene microplate wells, and incubated at 28 °C without agitation for 4 d. Biofilm production was examined after 2 and 4 d. At the corresponding time point, bacterial growth was determined by measurement of OD_600_. Then, the supernatant was removed from the wells, and biofilm remaining on the bottom of the wells was washed twice with 0.9% NaCl, and stained with 0.1% crystal violet. Next, biofilm was resolved by addition of 95% ethanol, and its amount was quantified by OD_560_ measurement in a microplate reader. For each time point, the experiment was repeated twice with three replicates for each strain analyzed, and data are presented as OD_560_/OD_600_.

### 2.13. Determination of Cell Topology and Properties Using Atomic Force Microscopy

Bacterial samples for atomic force microscopy (AFM) were prepared according to a method described earlier [60] with a minor modification [56]. Briefly, 6-h cultures of rhizobial strains were diluted in a fresh portion of the medium to OD_600_ = 0.1, centrifuged, and the bacterial pellets obtained were resuspended in 5 μL water, loaded onto 10-mm mica disks (Continental Trade, Warsaw, Poland), and allowed to dry in room temperature. Surface properties of rhizobial cells were imaged using a NanoScope V AFM (Veeco, Oyster Bay, NY, USA) in Analytical Laboratory, Faculty of Chemistry, Maria Curie-Skłodowska University, Lublin, Poland. All measurements (with the exception of DMT modulus; 5 N m^−1^ TAP150A, Bruker, Billerica, MA, USA), were done in the “Peak Force QNM” operation mode using a silicon tip with a spring constant of 96 N m^−1^ (NSG30, NT-MDT, Moscow, Russia). The following parameters were determined: height and peak force errors (cell topography), DMT (Derjaguin, Muller and Toporov) modulus (cell flexibility), adhesion (adhesion forces between the tip and the cell surface) and deformation (cell-surface stiffness). Data obtained were analyzed using Nanoscope Analysis ver. 1.40 software (Veeco, Plainview, NY, USA). Values of average rootmean-square (RMS) roughness were calculated using 20 fields sized 150 × 150 nm from 0.5 × 0.5 µm images of three individual bacteria, each from a different sample. A paired Student’s *t*-test was used to assess differences in tested parameters between the *pssZ* mutant and wild-type cells. The three-dimensional images and section profiles of the cells were generated using WSxM 5.0 software (Nanotec, Madrid, Spain) [61].

### 2.14. Plant Experiments

Symbiotic properties of rhizobial strains were determined using red clover (*Trifolium pratense* cv. Diana) as a host plant as described elsewhere [26]. Briefly, clover seedlings were placed on Fåhraeus slants [62] and after 4 days were inoculated with bacterial suspensions of OD_600_ = 0.2 (100 µL aliquot per plant). The plants were grown for 28 days under natural light supplemented with artificial light (14 h at 24 °C and 10 h at 18 °C) in a greenhouse, and nodules appearing on the roots were counted after each week. 4 week plants were harvested, and their wet shoots and roots were weighted. The experiment was done in triplicate using 20 plants for each strain tested.

### 2.15. Nodule Analysis Using Light and Electron Microscopy

To compare nodule occupation by the Rt297 mutant and the control strains Rt24.2, Rt297(pPL1) and Rt24.2(pPL1), the enzymatic activity of β-glucuronidase encoded by *gusA* in the mTn*5*SS*gusA*40 transposon or the pJBA21Tc plasmid was used [44]. Clover seedlings were inoculated with these strains and grown up to 4 weeks. Next, the nodules were stained using 50 mM sodium phosphate buffer (pH 7.2) containing 50 µg mL^−1^ of 5-bromo-4-chloro-3-indolyl-β-d-glucuronide [25] and analyzed under a Nikon light microscope (OPTIPHOT2). To characterize in detail the structure of the nodules elicited by the Rt297 and Rt24.2 strains, plant material was prepared for electron microscopy analysis as described earlier [25].

### 2.16. Statistical Analysis

The statistical analyses of data were performed using the Student’s *t*-test or the Statistica (ver.12, StatSoft, Cracov, Poland; one-way analysis of variance (ANOVA)) and significant differences between the analyzed samples for the Rt297 mutant and control strains were established at *p* < 0.05.

## 3. Results

### 3.1. Genetic Characterization of a Mutant Strain Rt297 and Complementation of a pssZ Mutation

**Figure 1:**
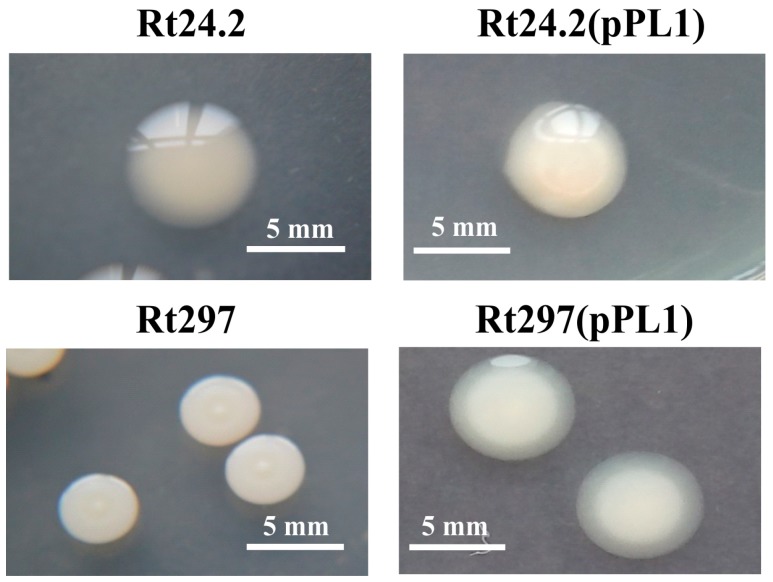
Morphology of colonies formed by the wild-type strain *R. leguminosarum* Rt24.2 and several derivatives: the mutant strain Rt297 (*pssZ*), complemented strain Rt297 (pPL1), and Rt24.2 (pPL1) harboring additional *pssZ* copies.

We recently analyzed the chromosomal Pss-I region of *R. leguminosarum* bv. *trifolii* Rt24.2 and determined its genetic organization [25]. Prior to that, a random mutagenesis of the Rt24.2 strain using an mTn*5*SS*gusA*40 transposon was performed to establish which of the genes from the Pss-I region affect EPS synthesis [42]. As a result, a few strains unable to produce EPS were obtained, among them Rt770 (*pssS*) and Rt1933 (*pssE*), which have been described previously [25,26]. In the current study, we characterized another Rt24.2 derivative, named Rt297, generated using the transposon mutagenesis described above [42]. This mutant strain formed small, non-mucoid colonies on 79CA agar plates, which essentially differed from those formed by the wild type (Figure 1).

**Figure 2:**
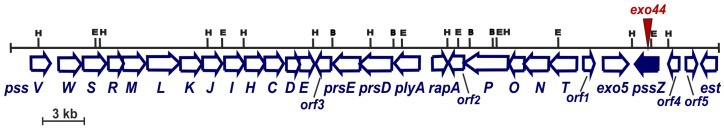
Physical and genetic map of the Pss-I region of *R. leguminosarum* Rt24.2. Arrows below the map indicate the direction of gene transcription. Selected restriction sites are marked: E, EcoRI; H, HindIII; and B, BamHI. Location of the mTn*5*SS*gusA*40 insertion in the mutant Rt297 genome is marked by a red triangle.

Using hybridization, PCR amplification with several primers complementary to different genes of the Pss-I region, and sequencing analyses, the localization of the mTn*5*SS*gusA*40 transposon in Rt297, designated the *exo44* mutation, in the *pssZ* gene was identified (between positions 1428 and 1429 nt, GenBank no. NZ_MAMO01000063) (Figure 2). We found that *pssZ* is an individual open reading frame (ORF) that does not form part of any operon; therefore, the mutation in this gene would not exert a polar effect on adjacent genes of the Pss-I region.

**Figure 3:**
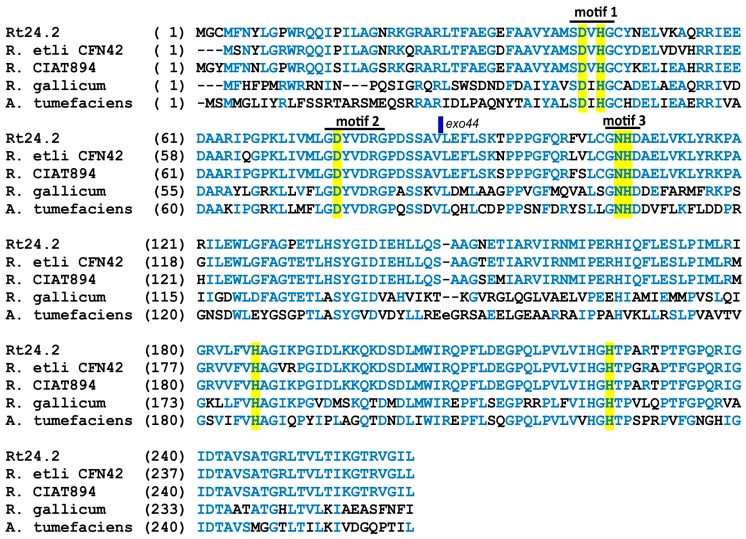
Alignment of amino acid sequences of STPs from *R. leguminosarum* Rt24.2 (PssZ) (GenBank WP_026230739.1), *R. etli* CFN42 (ABC92003.1), *R.* CIAT894 (WP_085738086.1), *R. gallicum* (WP_040115590.1), and *A. tumefaciens* (WP_012652475.1). Amino acids that are identical at individual positions in at least three of the analyzed proteins are marked by blue color. The exact point at which the PssZ protein is interrupted because of the *exo44* mutation in the *pssZ* gene (between residues 87 and 88) is marked by a blue rectangle. Motifs 1–3 are designated with black lines, whereas aa residues potentially engaged in metal binding are highlighted in yellow.

The presence of a single copy of mTn*5*SS*gusA*40 in the Rt297 genome was confirmed using Southern hybridization with a *gusA* probe (data not shown). The *pssZ* gene (locus BAE36_21610) encodes a 263-aa protein (the coding region extends from position 898 to 1689 nt, NZ_MAMO01000063), which shares high identity with bacterial serine/threonine protein phosphatases (STPs) belonging to the group of phosphoprotein phosphatases (PPP) from the metallophosphatase (MPP) superfamily (MPP_PPP family, Cd00144). Among rhizobia, PssZ of Rt24.2 (GenBank WP_026230739.1) shares high sequence identity with STPs of *R. etli* CFN42 (92% identity, GenBank ABC92003.1), *Rhizobium* sp. CIAT894 (94%, WP_085738086.1), *R. gallicum* (54%, WP_040115590.1), *Agrobacterium rhizogenes* (52%, WP_047457744.1), *A. tumefaciens* (50%, WP_012652475.1), *Mesorhizobium loti* (47%, WP_063898332.1), and *Bradyrhizobium lupini* (46%, EKJ95978.1). The mTn*5*SS*gusA*40 insertion in the Rt297 genome was identified 261 nt downstream of the 5′-end of the *pssZ* gene. Therefore, the encoded product is a truncated protein that lacks 176 C-terminal aa of the wild-type PssZ protein (Figure 3).

The PPP family is one of two known protein phosphatase families specific for serine and threonine. This family is ancient and its members are found in all eukaryotes, and in most bacteria and archaea [e.g., PP1, PP2A, PP2B (calcineurin), PP4, PP5, PP6, PP7, PrpE, PrpA/PrpB, and ApA4 hydrolase]. The catalytic domain of these PPP proteins usually contains three conserved motifs (-GDXHG-, -GDXVDRG-, and -GNHE-). We identified the catalytic domain at the N-terminus of the Rt24.2 PssZ (Figure 3), as well as sequences corresponding to the three conserved motifs. Among them, the first motif (-SDVHG-; identical aa are underlined) shared the lowest sequence similarity with the corresponding conserved motif (-GDXHG-), whereas the sequence of both the second (-GDYVDRG-) and the third (-GNHD-) motifs were almost identical to those of the conserved motifs (-GDXVDRG- and -GNHE-, respectively). Additional conserved aa residues (histidine, aspartate, and asparagine) were also detected in PssZ [at positions 43 (D), 45 (H), 76 (D), 107–108 (NH), 186 (H), and 225 (H)]. These aa are characteristic for enzymes belonging to the MPP family and are responsible for binding two metal ions (typically, manganese, iron, or zinc), which are coordinated by a double beta-sheet sandwich with a di-metal active site composed of residues located at the C-terminal region of the sheets.

In silico sequence analysis of a region upstream of *pssZ* revealed the presence of motifs with high identity with the −35 and −10 motifs of *E. coli* promoters, which are recognized by sigma^70^ RNA polymerase. This promoter sequence was located 376 nt upstream of the *pssZ* ORF (5′-TTGCCG-N_17_-TTTACT-3′; nucleotides identical with those of the *E. coli* promoter consensus are underlined). Based on PCR analyses using several primer pairs complementary to the promoterless *gusA* gene present in mTn*5*SS*gusA*40 and different *pssZ* regions, we have established that the *gusA* gene has the same transcriptional orientation as *pssZ*. Using a β-glucuronidase activity assay, we determined the transcriptional activity of the *pssZ* promoter to be 406.7 ± 56.8 Miller units.

**Figure 4:**
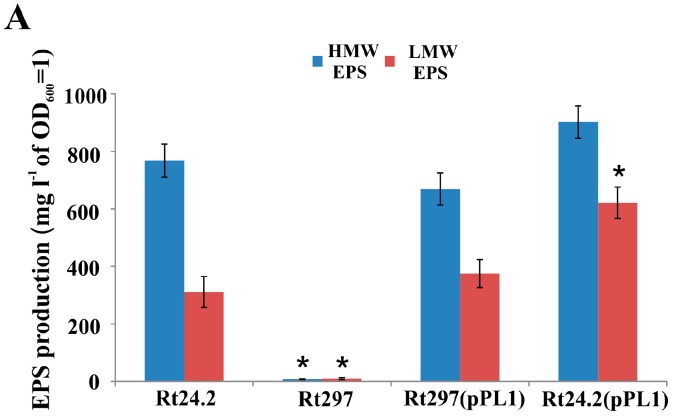

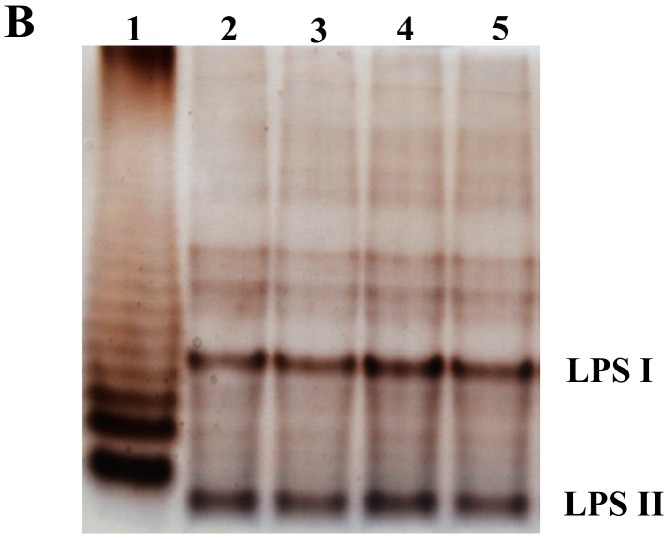
Production of high- (HMW) and low-molecular weight (LMW) exopolysaccharide EPS by wild-type strain *R. leguminosarum* Rt24.2 and several derivatives (**A**). The given values are averages ± SD of three independent experiments with three biological replicates for each strain analyzed. * The amount of EPS produced by the tested strains was significantly different from that produced by the wild-type strain Rt24.2 (*p* < 0.05, Student’s *t*-test). (**B**) Silver-stained Tricine SDS-PAGE profiles of LPS from Rt24.2 and several derivatives. The amount of LPS loaded into each well was 1 μg. Lanes: 1, LPS of *Salmonella enterica* sv. *Typhimurium* (Sigma, St. Louis, MO, USA); 2, LPS of Rt24.2; 3, LPS of Rt297; 4, LPS of Rt297(pPL1); and 5, LPS of Rt24.2(pPL1). LPS I, high-molecular weight LPS corresponding to smooth LPS; LPS II, low-molecular weight LPS corresponding to rough LPS.

In order to complement the *exo44* mutation of Rt297, plasmid pPL1, containing a 1.8-kb fragment that harbored the complete *pssZ* gene as well as its promoter region, was constructed. The introduction of plasmid pPL1 into the Rt297 mutant restored the mucoid colony phenotype (similar to that of the wild-type strain), indicating that this 1.8-kb fragment of the Pss-I region was sufficient to complement the *exo44* mutation. In addition, to establish the effect of the presence of additional *pssZ* copies on colony mucoidy, plasmid pPL1 was introduced into the wild-type strain Rt24.2. Next, the amounts of both high- and low-molecular weight (HMW and LMW, respectively) fractions of EPS produced by strains Rt24.2, Rt297, Rt297(pPL1), and Rt24.2(pPL1) cultured in 79CA medium with 1% glycerol as a carbon source were determined and compared (Figure 4A). Both control strains, Rt24.2 and Rt297(pPL1), produced large amounts of this polysaccharide, and HMW EPS was the dominant fraction (the HMW/LMW ratio for Rt24.2 was 2.33, whereas that for Rt297(pPL1) was 1.79). Moreover, Rt24.2(pPL1) synthesized more EPS (both HMW and LMW fractions) than the wild-type strain. The presence of empty vector pBBR1MCS-2 in Rt24.2 did not affect the level of EPS production, as confirmed elsewhere [39]. By contrast, Rt297 produced only residual amounts of EPS (1.09% of Rt24.2 HMW EPS and 3.48% of Rt24.2 LMW EPS), indicating that the synthesis of this polymer was dramatically impaired in this mutant. These data confirm that *pssZ* plays an essential role in EPS production in *R. leguminosarum*.

Furthermore, we asked whether the *exo44* mutation affected the synthesis of another surface polysaccharide, the lipopolysaccharide (LPS), in Rt297. Consequently, LPS was extracted from Rt24.2, Rt297, Rt297(pPL1), and Rt24.2(pPL1), and analyzed by SDS-PAGE. As shown in Figure 4B, the LPS electrophoretic profiles of these strains were identical, suggesting no changes in this polymer. However, we cannot exclude the possibility that the mutation in *pssZ* resulted in minor changes in the structure of LPS that did not affect the electrophoretic mobility of this polysaccharide.

### 3.2. The exo44 Mutation Negatively Affects Growth and Motility of Rhizobium leguminosarum

**Figure 5:**
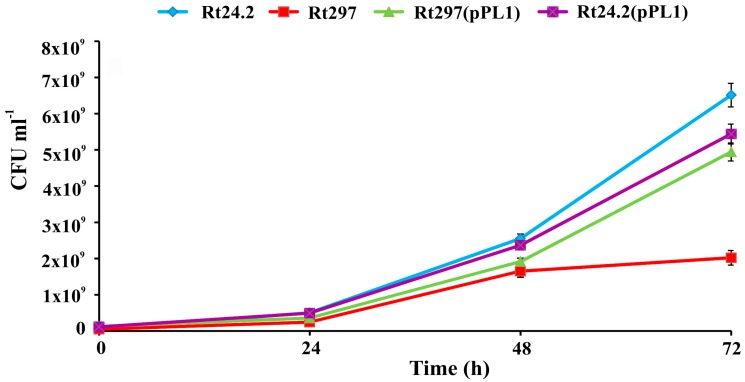
Growth kinetics of the wild-type strain *R. leguminosarum* Rt24.2 and its derivatives in 79CA medium. The given values are averages ± SD of two independent experiments with three biological replicates for each strain analyzed.

Next, we checked whether the *pssZ* mutation affected other physiological properties of *R. leguminosarum*. First, growth kinetics of Rt297 was compared with those of the Rt24.2, Rt297(pPL1), and Rt24.2(pPL1) strains in a rich 79CA medium during a 72-h experiment (Figure 5). Since the different amounts of EPS produced by these strains could putatively influence the culture optical density readings, CFU mL^−1^ values were determined instead.

The growth rate of Rt297 was considerably slower than that of strains Rt24.2, Rt297(pPL1), and Rt24.2(pPL1). After 72 h, the final number of viable cells of the *pssZ* mutant was about three-fold lower than that of the wild-type strain Rt24.2. The growth of Rt297(pPL1) and Rt24.2(pPL1) was slower than that of the wild type, which was most probably caused by the presence of kanamycin in the growth medium of the former strains. The generation time was also determined for all these strains. The experiment revealed that Rt24.2, Rt24.2(pPL1), and Rt297(pPL1) had similar doubling times (Rt24.2: 4.5 ± 0.5 h; Rt24.2(pPL1): 5.0 ± 0.25 h; and Rt297(pPL1): 5.25 ± 0.5 h). In contrast, the generation time of Rt297 cells (6.75 ± 0.25 h) was significantly longer (*p* < 0.05; Student’s *t*-test) than that of the wild-type cells. These observations indicate that the *exo44* mutation negatively impacts the growth of *R. leguminosarum* cells.

In order to avoid any problem related to different growth kinetics of the tested strains, in next set of experiments we used bacterial cultures and suspensions with similar optical densities (OD_600_).

**Figure 6:**
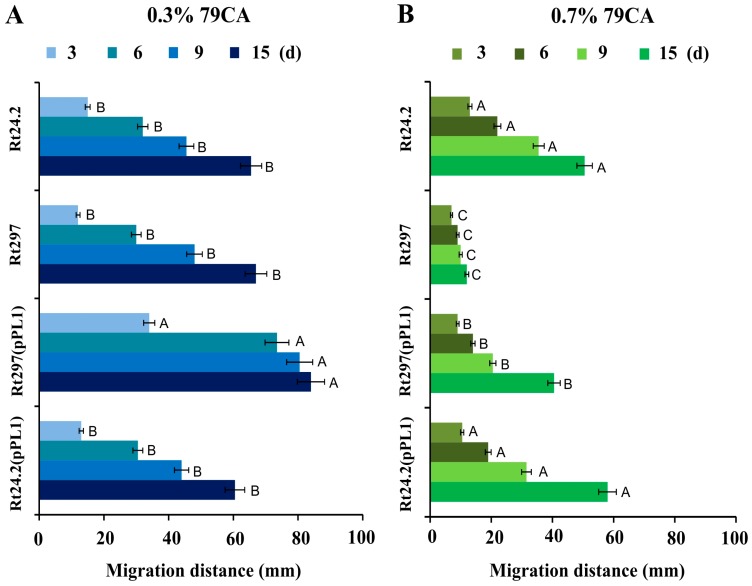
Motility of the wild-type strain *R. leguminosarum* Rt24.2 and its derivatives tested in 0.3% (**A**) and 0.7% 79CA (**B**) media for 15 d. The given values are averages ± SD of two independent experiments with three biological replicates for each strain tested. Statistically significant differences between the studied strains at the individual time points tested are designated with different letters (*p* < 0.05, one-way analysis of variance (ANOVA).

Next, we examined the motility of the *pssZ* mutant and wild-type cells during a 15 d experiment (Figure 6). The migration ability of the studied strains was tested using the 79CA medium containing 0.3% agar (swimming motility experiments) or 0.7% agar (surface motility experiments). The motility of Rt24.2, Rt24.2(pPL1), and Rt297(pPL1) was similar. The migration distance for these strains was larger in the medium containing 0.3% agar (Figure 6A) than in that containing 0.7% agar (Figure 6B). Although swimming capacity of Rt297 was not affected (Figure 6A), the mutant exhibited reduced surface motility on 0.7% agar (from 2- to 4-fold, depending on the time-point evaluated in comparison with Rt24.2) (Figure 6B). The largest difference in cell migration between Rt297 and Rt24.2 strains (~4-fold) was observed after 15 days of incubation. These data indicate that the *exo44* mutation affects surface motility of *R. leguminosarum* cells.

### 3.3. The exo44 Mutation Affects Surface Properties of Rhizobium leguminosarum Cells

Since EPS is an important component of the rhizobial cell envelope, we examined whether the *pssZ* mutation affected different cell-surface properties. First, the sensitivity of Rt24.2, Rt297, Rt297(pPL1), and Rt24.2(pPL1) strains to stress factors, such as SDS, DOC, and ethanol, was determined. The control strains, Rt24.2 and Rt297(pPL1), exhibited similar tolerance to the tested factors (Table 2). By contrast, the Rt297 mutant exhibited a slightly increased sensitivity to these stressors, although only in the case of ethanol the difference was statistically significant. Moreover, Rt24.2(pPL1) (the strain harboring additional *pssZ* copies) exhibited greater tolerance to the tested stressors than the wild-type strain Rt24.2 (statistically significant differences between these strains for SDS and DOC).

**Figure 7:**
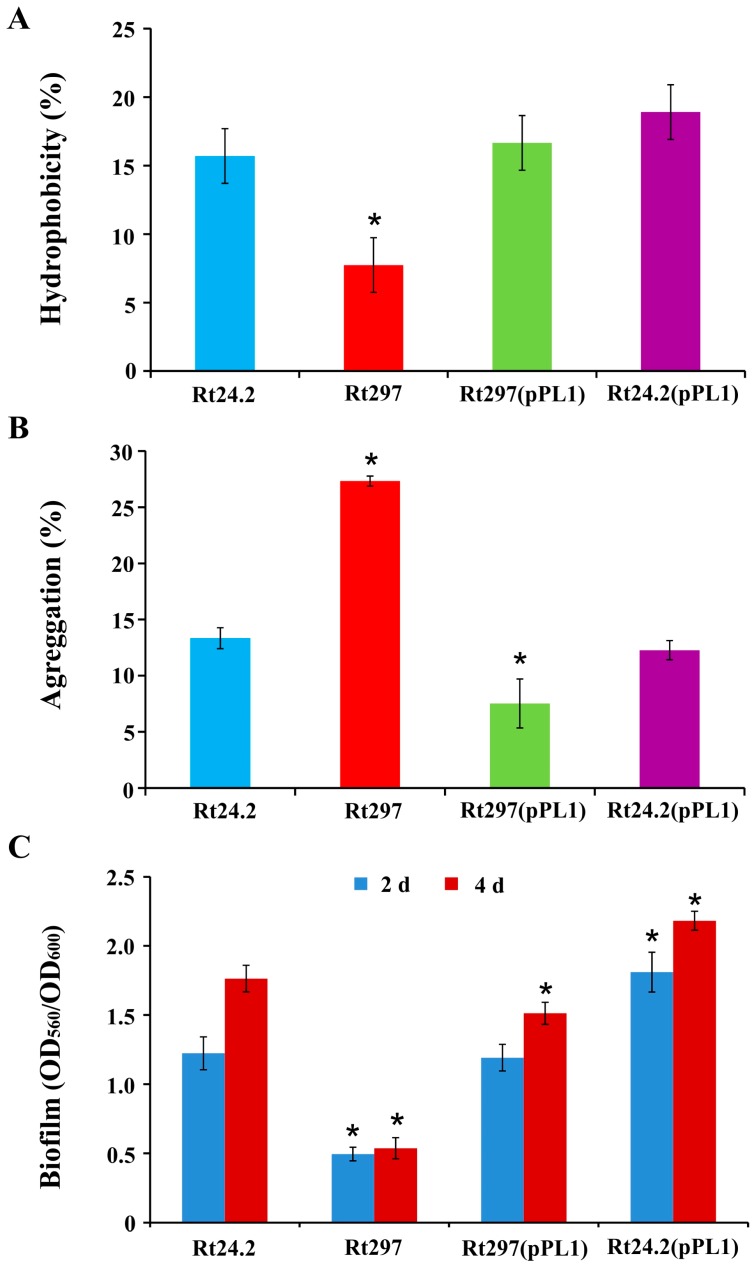
Cell hydrophobicity (**A**), aggregation ability (**B**), and biofilm formation (**C**) by the wild-type strain *R. leguminosarum* Rt24.2 and its derivatives. The values are averages ± SD of two independent experiments with three biological replicates for each strain analyzed. * Significant difference between Rt24.2 and the remaining strains submitted to a given treatment (*p* < 0.05, Student’s *t*-test).

We also examined cell hydrophobicity of the tested strains using bacterial cultures and a two-phase method with dodecane. In this assay, the hydrophobicity values for the wild-type Rt24.2 and the complemented *pssZ* mutant Rt297(pPL1) strains were similar (Figure 7A). The hydrophobicity of the Rt24.2(pPL1) cells was slightly higher than that of the control strains. By contrast, Rt297 cells exhibited a nearly 2-fold lower hydrophobicity than Rt24.2 and Rt297(pPL1) cells.

The aggregation ability of these strains was also examined (Figure 7B). Rt24.2, Rt297(pPL1), and Rt24.2(pPL1) cells showed similar tendency to aggregate. By contrast, the mutant Rt297 cells aggregated more effectively than the control cells.

Next, the amount of biofilm formed by the strains after a 2- or 4-d incubation was determined and compared (Figure 7C). The control strains Rt24.2 and Rt297(pPL1) produced large amounts of biofilm, although on day 4, the latter produced 11% less biofilm than the former. Rt24.2(pPL1) was even slightly more effective in biofilm formation than the wild type (on day 4 it produced 28% more biofilm than Rt24.2). By contrast, the Rt297 mutant produced significantly smaller amounts of biofilm during this period of time than the other strains tested (with a 3.1- to 4.2-fold reduction). The formation of these amounts of biofilm by the *pssZ* mutant was probably caused by the synthesis of residual amounts of HMW and LMW EPS. However, apart from EPS, other bacterial components, such as cell-surface-associated proteins (e.g., agglutinins), are also engaged in this process [3], and might account for the biofilm formed by the *pssZ* mutant.

**Figure 8:**
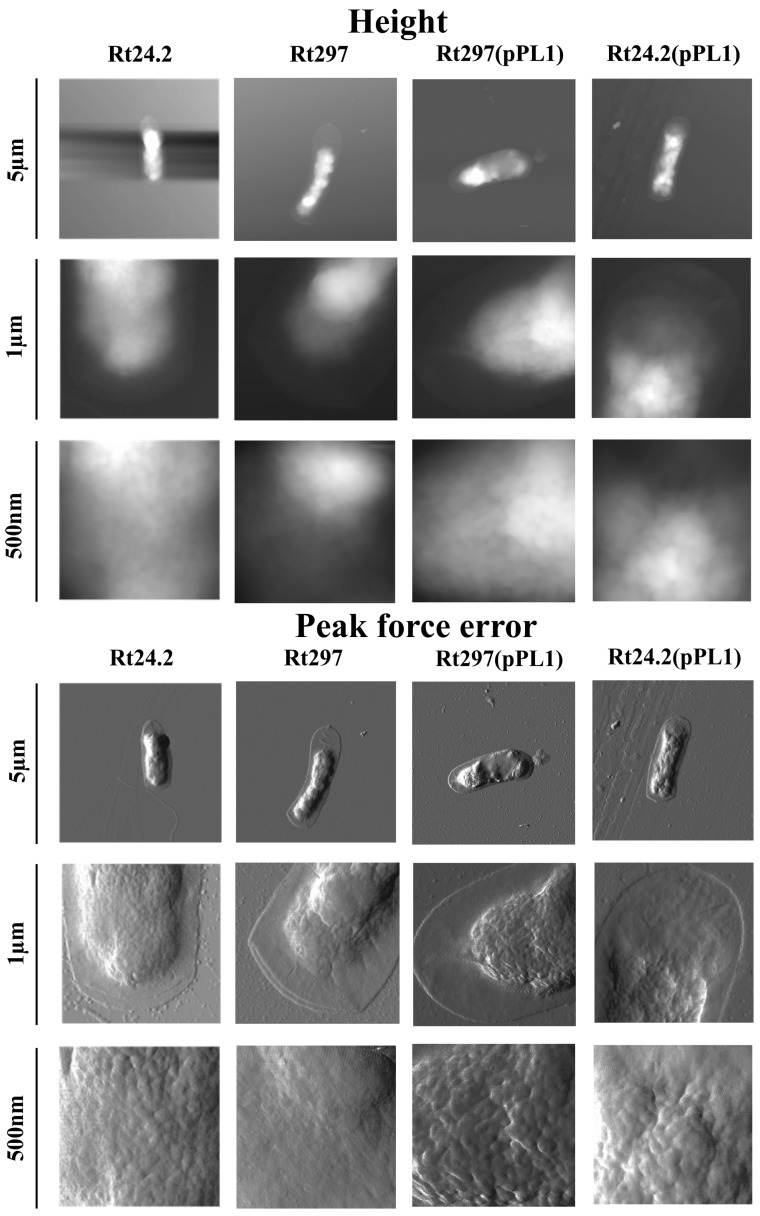
AFM imaging of the wild-type strain *R. leguminosarum* Rt24.2 and its derivatives Rt297, Rt297(pPL1), and Rt24.2(pPL1). The height and peak force error images are presented. The brighter and darker image areas correspond to the higher and lower parameter values, respectively.

**Figure 9:**
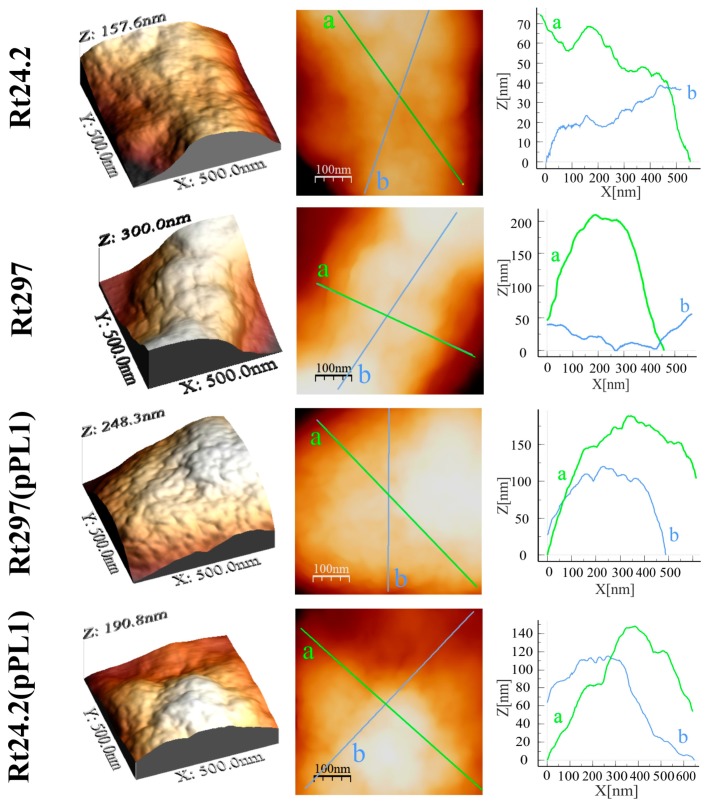
Atomic Force Microscopy (AFM) imaging of the wild-type strain *R. leguminosarum* Rt24.2 and its derivatives Rt297, Rt297(pPL1), and Rt24.2(pPL1). 3D images, height images, and section profiles corresponding to lines in the height images are presented (green and blue lines correspond to section profiles done in various planes of the cells).

In order to characterize in more detail cell-surface properties of the *pssZ* mutant, atomic force microscopy (AFM) imaging of Rt24.2, Rt297, Rt297(pPL1), and Rt24.2(pPL1) cells was performed (Figure 8 and Figure 9, and Table 3).

This analysis revealed some differences in topography and surface properties of Rt297 and control cells. Rt24.2, Rt297(pPL1), and Rt24.2(pPL1) cells were rod-shaped, with regularly spaced, small granules and shallow grooves on the envelope surface, and with similar average length and width (Table 3). By contrast, Rt297 cells were slightly bigger and had a more irregular shape, as seen in the height and peak force error images (Figure 8).

Further, the surface of Rt297 cells was smoother and less granular than that of the wild-type cells (Figure 8 and Figure 9). Nanomechanical properties of the mutant cells were also altered in relation to the wild type, as shown in the peak force error images. Apart from these visually apparent properties, differences in calculated values corresponding to the cell surface roughness, elasticity, and adhesion were determined. The roughness of the mutant cell surface was 3-fold lower than that of the wild-type cells (Table 3), whereas a nearly 2-fold increase in DMT modulus was observed. This indicated that the surface of the mutant cells was smoother and more inflexible than that of the wild-type cells. Generally, the higher the DMT modulus, the lower the elasticity of bacterial cell surface. Finally, the adhesion of Rt297 cells (determined as the adhesion forces between the AFM tip and bacterial cell surface) was nearly 2-fold weaker than that of the wild-type strain. For all the properties examined, the presence of plasmid pPL1 restored the determined Rt297 parameters to those of the wild-type strain. Collectively, these data indicate that the *pssZ* mutation affects the morphology and surface properties of *R. leguminosarum* cells.

### 3.4. The exo44 Mutation Leads to Disturbances in Symbiosis of Rhizobium leguminosarum with Clover

**Figure 10:**
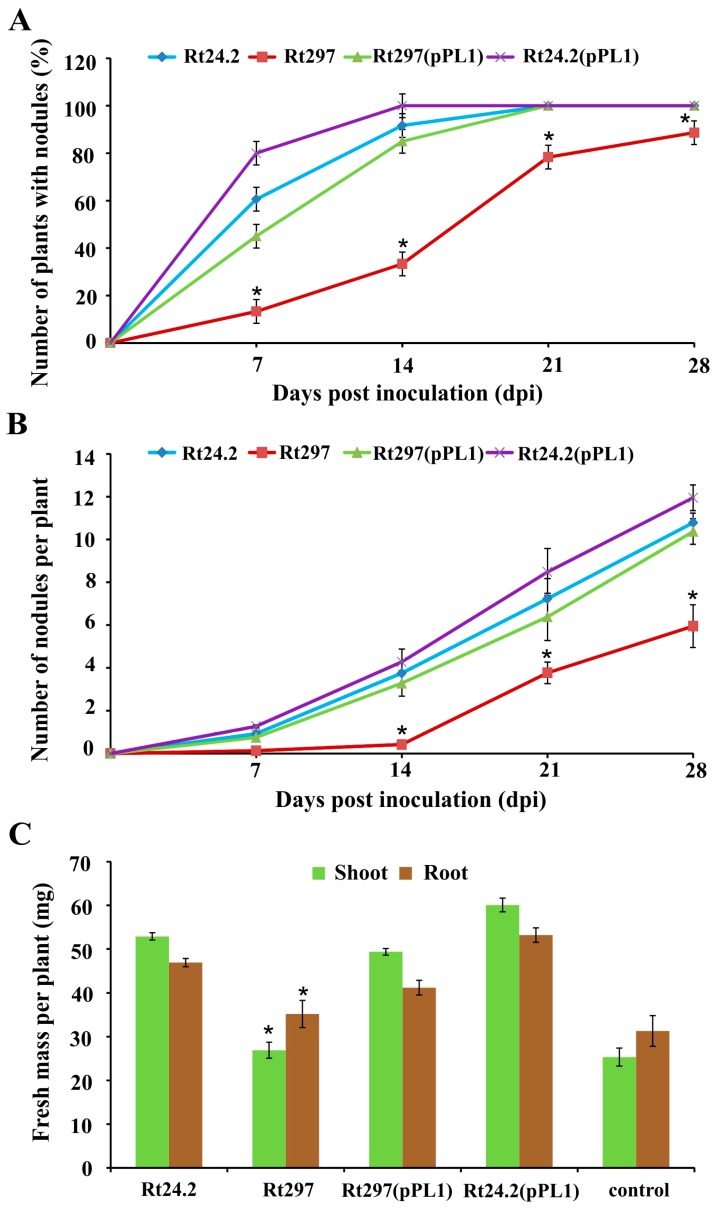
Effectiveness of clover root infection by the wild-type strain *R. leguminosarum* Rt24.2 and several derivatives presented as the percent of tested plants forming nodules (**A**); the number of nodules elicited by the studied strains on clover roots (**B**); and the shoot and root masses of 28-dpi clover plants inoculated with the different strains (**C**). Control, uninoculated clover plants. The values are averages ± SD of three independent experiments using 20 plants for each strain tested. * Significant differences between the Rt297 mutant and the wild-type Rt24.2 strain (*p* < 0.05, Student’s *t*-test).

We then investigated whether the observed changes of phenotypic properties of the Rt297 mutant affected its symbiotic interaction with clover. To this end, red clover (*Trifolium pratense*) seedlings were inoculated with strains Rt24.2, Rt297, Rt297(pPL1), and Rt24.2(pPL1), and grown for 4 weeks in a greenhouse (Figure 10).

**Figure 11:**
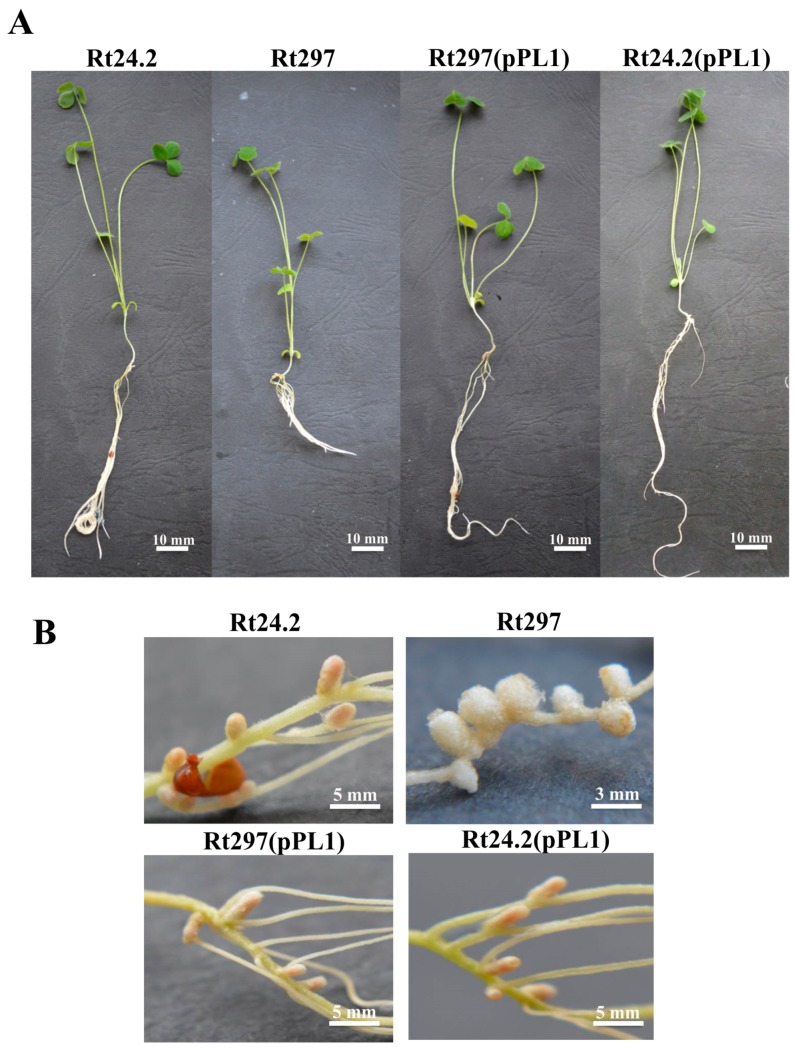
Clover plants at 28 dpi with the wild-type strain *R. leguminosarum* Rt24.2 and its derivatives (**A**) and nodules elicited on their roots by these strains (**B**).

The strains Rt24.2, Rt297(pPL1), and Rt24.2(pPL1) exhibited high effectiveness in host root infection, and after 21 days post inoculation (dpi) all plants inoculated with these bacteria (100%) had developed nodules on their roots (Figure 10A). In contrast, the capacity of the *pssZ* mutant to infect clover roots was severelly reduced, as evidenced by only 10% and 30% plants containing root nodules at 7 dpi and 14 dpi, respectively. Apart from a delay in nodule formation, the total number of nodules induced by Rt297 on the host roots at 28-dpi was considerably lower (~2-fold) than those elicited by strains Rt24.2, Rt297(pPL1), and Rt24.2(pPL1) (Figure 10B). Moreover, plants inoculated with the mutant Rt297 were miniscule and only formed small, white nodule-like structures, often with atypical shape, whose morphology suggested that they were ineffective in nitrogen fixation (Figure 11A,B). This was confirmed by the shoot mass of plants inoculated with Rt297, which was nearly 2-fold lower than the shoot mass of plants inoculated with the control strains, and very similar to that of the uninoculated plants (Figure 10C). In contrast, clover plants inoculated with Rt24.2, Rt297(pPL1), and Rt24.2(pPL1) were tall, with many pink elongated nodules on their roots, which suggested that they were effective in nitrogen fixation (Figure 11A,B).

**Figure 12:**
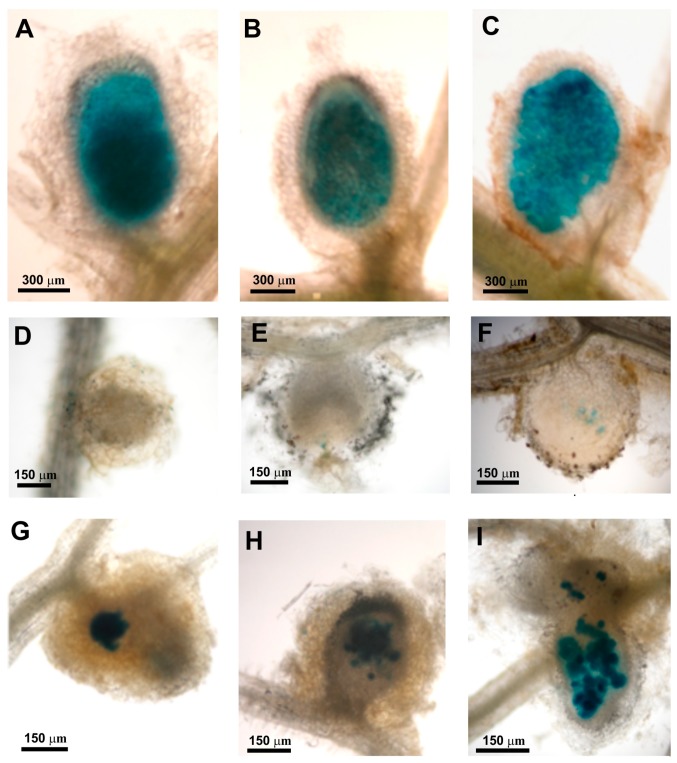
Light microscopy of clover (*Trifolium pratense*) root nodules elicited by the wild-type strain *R. leguminosarum* Rt24.2 and several derivatives harboring *gusA* reporter gene encoding β-glucuronidase. The images show 21-dpi nodules occupied by Rt24.2 (**A**), Rt297(pPL1) (**B**), and Rt24.2(pPL1) (**C**). (**D**–**I**) Nodules formed after inoculation with Rt297: (**D**–**F**) 7–dpi nodules; (**G**,**H**) 14-dpi nodules; and (**I**) a 21-dpi nodule.

Next, the occupation of clover root nodules by these rhizobial strains was examined. For this experiment, bacteria harboring the *gusA* gene encoding β-glucuronidase were used (Figure 12). We observed that strains Rt24.2, Rt297(pPL1), and Rt24.2(pPL1) effectively occupied the nodules, and these bacteria were detected in all zones of the 21-dpi nodules (i.e., infection zone, interzone, and nitrogen-fixing zone), with the exception of the meristem (Figure 12A–C). In contrast, occupation of clover root nodules by Rt297 was considerably reduced. Mutant cells were visible mainly on the root and nodule surface, and a great majority of the nodules were not occupied by this bacterium (Figure 12D,E). Rt297 cells were found inside single nodule cells only sporadically (Figure 12F–H). Even in such sporadic older (21-dpi) nodules, bacteria were present only in a few plant cells (Figure 12I).

**Figure 13:**
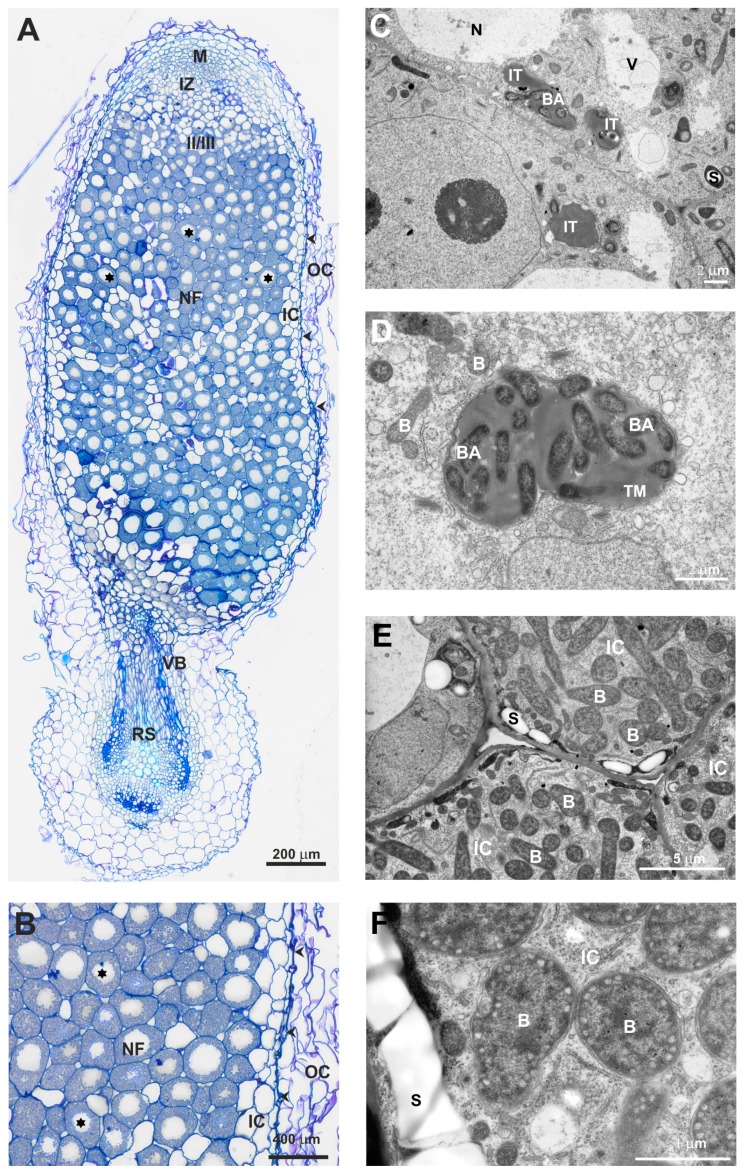
Semi-thin section of a 21-dpi clover root nodule elicited by the wild-type strain *R. leguminosarum* Rt24.2 (**A**), OC: outer cortex; IC: inner cortex; RS: root stela; VB: nodule vascular bundle; M: meristem; IZ: infection zone; II/III: interzone; NF: nitrogen fixation zone; black asterisks: infected cells; arrowheads: nodule endodermis. (**B**), nitrogen fixation zone with mature infected plant cells; NF: nitrogen fixation zone; OC: outer cortex; IC: inner cortex; black asterisks: infected cells; arrowheads: nodule endodermis. (**C**,**D**), Ultrastructure of an infection thread (IT) containing Rt24.2 cells; TM: thread matrix; BA: bacteria; B: bacteroids; S: starch granules. (**E**,**F**), Mature infected cells; IC: infected cell; B: bacteroids; S: starch granules.

Next, we characterized the structure of nodules elicited by the Rt297 mutant on clover roots in more detail. We previously described the structure of wild-type nodules induced by the Rt24.2 strain on this host plant [25,56]. As shown on Figure 13, wild-type clover nodules exhibit a typical structure with all zones characteristic for indeterminate-type nodules, including a large nitrogen fixation (NF) zone with numerous mature infected plant cells containing properly differentiated bacteroids (Figure 13A,B,E,F). ITs exhibited a normally formed thread wall and large amounts of thread matrix (Figure 13C,D).

**Figure 14:**
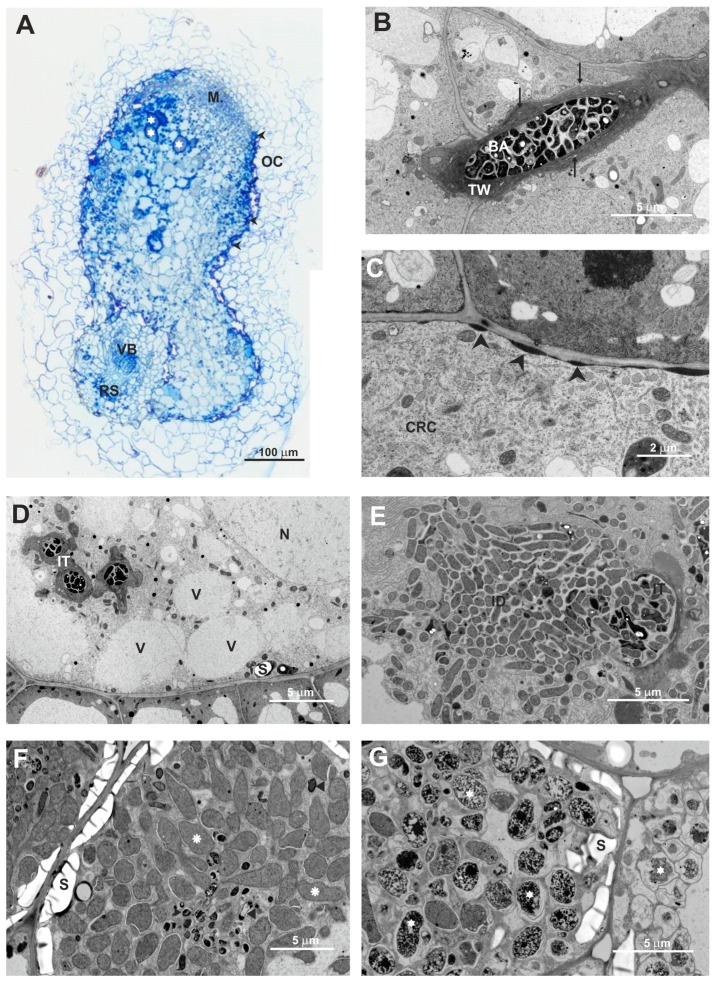
Semi-thin section of a 21-dpi clover root nodule elicited by the *R. leguminosarum* mutant Rt297 (**A**), M: meristem; OC: outer cortex; RS: root stela; VB: nodule vascular bundle; white asterisks: infected cells; arrow head: nodule endodermis. (**B**), Ultrastructure of the IT containing Rt297 cells: BA: bacteria, TW: thread wall; arrows: material deposited between osmiophilic layers of the thread wall. (**C**), Deposits of electron-dense material on the cortical root cell wall (arrow heads) (CRC: the cortical root cell). (**D**), A cortical root cell which undergoes autolysis; N: nucleus; IT: infection thread; S: starch granules; V: vacuoles. (**E**), “Explosion-like” mass endocytosis of bacteria by the plant cell cytoplasm; ID: infection droplet. (**F**), A mature infected cell; S: starch granules; rosette: abnormally differentiated bacteroids; triangle: precociously degenerated bacteroids. (**G**), an infected cell containing degrading bacteroids (stars); S: starch granules.

Similarly with wild-type nodules, nodules elicited by Rt297 (Figure 14) were surrounded by a cortex containing large, loosely arranged cells and by an endodermis. However, in contrast with the wild-type nodules, semi-thin sectioning of the mutant nodules revealed that a great majority of their central tissue contained uninfected parenchymatous cells with starch grains and only few of these cells were infected by bacteria (Figure 14A). The nodules contained the meristem and the vascular bundle connected with the root stele, root epidermal cells (in contact with the Rt297 cells) had thickened walls, which stained intensely with azur A and methylene blue. The formed ITs were wide and often branched, with many mutant cells tightly packed inside them; the shape and size of these bacteria were highly variable (Figure 14B). IT walls were atypically thick, irregularly formed, with knobs and protrusions (Figure 14C). Another altered morphological feature of these mutant-induced ITs was the lack of thread matrix (Figure 14D), which is typically present in large amounts within the wild-type ITs. Plant cell infection was also abnormal; it suggested a simultaneous endocytosis of many mutant cells into the plant root cell cytoplasm, termed “explosion-like” mass endocytosis (Figure 14E). Although symbiosomes formed in these cells usually contained only a single bacteroid, and those with more than one bacteroid were found only sporadically (similarly to wild-type nodules) (Figure 14F), differentiation of the mutant bacteroids was essentially different from that of the wild-type bacteroids and several disturbances of this process were observed. Only some bacteroids differentiated, while a great majority of bacteroids degenerated precociously. These bacteroids were larger than normal, abnormally swollen, often deformed, and underwent rapid degradation. As a consequence of deterioration, their homogenous cytoplasm became electron-dense, marbled, or completely dark (black) (Figure 14G).

In conclusion, all these data indicate that the lack of PssZ leads to pronounced disturbances in the symbiosis between Rt297 and clover, both in early (i.e., host root infection) and late (i.e., bacteroid development) steps. The mutant infected only few nodule cells and the bacteroids degenerated prematurely, resulting in the inability of these nodules to fix nitrogen.

## 4. Discussion

In the current study, we showed that inactivation of the *R. leguminosarum* bv. *trifolii pssZ* gene results in a dramatic impairment in EPS production. Cell growth and motility of the *pssZ* mutant was also affected, with the cells presenting altered surface properties and clearly impaired in symbiotic interaction with clover. Most probably, at least some of these alterations were caused by the lack of EPS production, as has been previously demonstrated by our group for various *R. leguminosarum* bv. *trifolii* mutants with impaired biosynthesis of this surface polysaccharide [17,26,56,63]. As recently reported [13,14,15], rhizobial EPS plays a crucial role as a signal molecule in the early stages of symbiosis. Furthermore, this polysaccharide is important for adhesion and biofilm formation on both abiotic surfaces and plant roots, as well as for the protection of bacterial cells against several environmental conditions [17,64,65].

The synthesis of rhizobial EPS is a multi-step process which involves the activity of many enzymes and regulatory proteins. A great majority of proteins that participate in this biosynthetic pathway in *R. leguminosarum* are encoded by genes from the large chromosomal cluster Pss-I [24,25,66,67]. To date, the only exception is *pssA*, located a long distance away from the Pss-I region [6,9]. This gene encodes an enzyme that initiates the EPS synthesis. PssA transfers glucose-1-phosphate from UDP-glucose to the lipid anchor und-PP in the bacterial IM [23]. Earlier studies showed that mutations in several genes encoding glycosyltransferases involved in the addition of sugar residues to the growing EPS subunit abolish EPS production. This was confirmed by the EPS-deficient phenotypes of *R. leguminosarum* strains harboring mutations in *pssA*, *pssDE*, *pssC*, and *pssS*, which encode glycosyltransferases participating in the first four steps of the EPS subunit assembly [6,7,9,24,25,26]. Moreover, the *R. leguminosarum exo*344::Tn*5* strain, carrying a mutation in *pssJ*, which encodes a protein involved in the last step of the subunit assembly, synthesized only residual amounts of EPS, whose units lack the terminal D-galactose [21,22]. Similarly, mutations in genes responsible for the synthesis of sugar nucleotide precursors exert strong negative effects on this process. For example, a mutation in the *exo5* gene located adjacent to *pssZ* in the Pss-I cluster results in pleiotropic effects. The *exo5* mutant strain of *R. leguminosarum* RBL5523 is defective in UDP-glucose dehydrogenase, which converts UDP-glucose to UDP-glucuronic acid [68,69]. This mutant is unable to synthesize two sugar precursors, UDP-glucuronic acid and UDP-galacturonic acid. Consequently, it is unable to synthesize EPS and capsular polysaccharide, its LPS lacks galacturonic acid, and is defective in symbiosis with its host plant, *Vicia sativa* subsp. nigra. Similar effects have been described for the *S. meliloti* and *S. fredii* mutants affected in the *exo5* orthologue, *rkpK* [70,71]. In both rhizobia, the *exoK* mutants produce altered LPS, although the EPS production is only affected in the *S. fredii rkpK* mutant because of the presence of glucuronic acid in *S. fredii* EPS.

Mutation of *pssZ*, which is located adjacent to *exo5*, also abolished EPS synthesis in *R. leguminosarum*, indicating that the protein encoded by *pssZ* plays an essential role in this process. However, in spite of similarities between the pleiotropic effects of mutations in the *pssZ* and *exo5* genes, the *exo44* mutation most probably does not directly affect the *exo5* expression since these two genes are transcribed in opposite directions. It seems more probable that the protein product of *pssZ* functions at a post-translational level, directly and/or indirectly affecting enzymatic activity of some protein/proteins involved in EPS synthesis. Moreover, the high sequence identity shared by PssZ and bacterial STPs suggests that the function of this protein in *R. leguminosarum* might be more global that simply influencing the EPS biosynthetic pathway (being an element of rhizobial signal regulatory cascade). To the best of our knowledge, this is the first-ever report regarding the characterization of a protein of this type in rhizobia. We showed that the *pssZ* mutation not only inhibited EPS synthesis, but also negatively affected bacterial behavior and cell-surface properties. This was observed as significantly reduced growth kinetics (leading to increased generation time), surface motility, cell hydrophobicity, and biofilm formation, and altered cell morphology and surface properties. The ability of Rt297(*exo44*) to interact with clover was dramatically impaired most probably because of the absence of EPS production: the reduced ability to infect clover roots led to the delayed formation of a reduced number of non-properly infected nodules that were inefficient in nitrogen fixation. The introduction of plasmid pPL1 harboring the *pssZ* gene into Rt297 restored the mucoid colony phenotype and other bacterial properties to values that were similar to those of the wild-type cells (although some characteristics such as growth, biofilm formation, and nodulation effectiveness parameters were slightly lower than those for Rt24.2). On the other hand, although Rt24.2(pPL1) harboring additional *pssZ* copies also grew slightly slower than the wild type, most probably because of the presence of the harbored vector and antibiotic in the culture medium, this strain exhibited higher EPS and biofilm production, increased tolerance to some stress factors, and enhanced symbiotic effectiveness than Rt24.2.

A central question in bacterial physiology is how bacteria sense and respond to their environment. In general, two-component signaling systems, composed of a sensor protein histidine kinase that activates a transcription factor response regulator in response to a specific signal, play a dominant role in bacterial signaling [72]. However, bacteria also possess signaling systems composed of eukaryotic-like Ser/Thr kinases (STKs) and STPs. Even though these systems do not have dedicated transcription factors, they are capable of affecting gene expression [73,74,75]. Previously, regulatory Ser/Thr phosphorylation has been assumed to be largely absent in prokaryotes. However, based on recent phosphoproteomic analyses, numerous (~70) proteins with phosphorylated Ser or Thr residues were identified in both Gram-positive and Gram-negative bacteria [76,77]. Among them, were several proteins of catalytic, transporting, and regulatory functions, related to different processes (replication, transcription, translation and posttranslational modifications; transport and metabolism of amino acids, carbohydrates, and inorganic ions; polysaccharide synthesis) [78,79,80]. As was shown for *Bacillus subtilis*, a Gram-positive model bacterium widely used in both basic research and industrial applications, Ser/Thr kinase PrkC and phosphatase PrpC are involved in spore development and biofilm formation [81,82]. PrkC and PrpC phosphorylates and dephosphorylates oxidoreductase YkwC, respectively, and Ser281 phosphorylation abolishes the activity of this enzyme. In the case of *Staphylococcus aureus*, a role of STK and STP in modulation of cell wall structure was confirmed [83]. Similarly, STK and STP enzymes affect growth, cell segregation, and virulence of *Streptococcus agalactiae* and *S. pyogenes* [84,85]. In contrast, considerably less data are available for these proteins in Gram-negative bacteria, although the presence of STK and STP proteins has been described in *Pseudomonas aeruginosa* [86]. All these observations confirm the notion that protein phosphorylation is important in controlling a variety of biological processes in bacteria, such as cell differentiation and proliferation, metabolism, and cell wall biogenesis.

In general, STPs belong to PPP from the MPP superfamily (MPP_PPP family, Cd00144). The PPP family is ancient with members found in all eukaryotes, and in most bacterial and archeal genomes [73,74,75]. The catalytic domain of these PPP usually contains three conserved motifs (-GDXHG-, -GDXVDRG-, and -GNHE-). The presence of these motifs was identified in the amino acid sequence of PssZ protein, characterized in this study. The MPP superfamily contains functionally diverse enzymes but all share a conserved domain with an active site consisting of two metal ions (usually, manganese, iron, or zinc) coordinated with octahedral geometry by a cage of histidine, aspartate, and asparagine residues [73,74,75]. STPs are classified into two distinct structural families: PP1/PP2A/PP2B, and PP2C, according to their substrate specificity, metal-ion dependence, and sensitivity to phosphatase inhibitors, the phosphatase catalytic subunits, and isoforms [87]. PP1 and PP2A are active independently of the presence of metal ions, whereas PP2B is Ca^2+^-calmodulin dependent and PP2C is metal-dependent [88,89,90]. A few histidine, aspartate, and asparagine residues as target sites for metal-binding activity were identified in the PssZ sequence, suggesting that this protein belongs to the PP2C-type family. Among bacterial homologs of eukaryotic-type Ser/Thr phosphatases, several enzymes, such as SppA in *Streptomyces coelicolor* and Pph1 in *Micococcus xanthus*, are PP2C-type STPs that are important for vegetative growth and development of these bacteria [91,92]. Similarly to our observations for the *R. leguminosarum pssZ* mutant, it was reported that mutations inactivating eukaryotic-type Ser/Thr kinase (Stk1) and phosphatase (Stp1) exhibit pleiotropic effects on growth, cell segregation, and virulence of *S. agaliactae*, suggesting an important role for these enzymes in the regulation of various cellular processes in this bacterium [90].

Thus, recent studies have shown that prokaryotes contain signaling enzymes commonly found in eukaryotes, such as STKs and STPs, which contribute to the regulation of gene expression that is important for several cellular processes, such as growth, virulence, secondary metabolite production, and cell envelope biogenesis [93]. Although they are not DNA-binding proteins, STKs and STPs mediate prokaryotic gene expression through post-translational modification of a variety of targets, including two-components response regulators or critical components of the prokaryotic transcriptional and translational machinery [94].

In relation to the deficiency of EPS production exhibited by the *R. leguminosarum pss*Z mutant, most probably it could be associated with the absence of modulation of the activity of some Pss proteins involved in EPS synthesis. Many different proteins could constitute targets for PssZ action, including enzymes involved in sugar precursor synthesis, glycosyltransferases, proteins engaged in EPS polymerization and export, and/or regulatory proteins. The protein product of the *exo5* gene might be a target for PssZ, because of the high similarity of the pleiotropic effects of mutations in *exo5* and *pssZ*. Moreover, PssA, which plays an essential role in the formation of the enzymatic machinery in the bacterial IM and initiates the EPS biosynthetic process, could also be a target for this type of regulation. In fact, this protein contains an unusually high number of Ser and Thr residues [9]. Recently, Marczak and others [4] postulated that the mechanisms of EPS synthesis and translocation out of the cell must be temporally and spatially coordinated. Further, direct interactions between proteins of this multi-protein complex must occur for their effective activity, in which phosphorylation/dephosphorylation reactions must play crucial roles. In addition, the possibility that PssZ could modulate the activity of other proteins not related to EPS biosynthesis cannot be discarded. Sequence identity shared by PssZ and STPs, which play an important role in bacterial signaling, suggests that the function of this protein in *R. leguminosarum* might be global and influences other cellular processes in addition to participating in the EPS biosynthesis. Clearly, further and comprehensive research at the transcriptomic and proteomic levels is needed to clarify this issue.

We have identified three genes (BAE36_16215, BAE36_06965, and BAE36_31125) in the Rt24.2 genome (acc. nos. MAMO01000032, MAMO01000009, and MAMO01000168) coding for proteins (150 aa, 247 aa, and 697 aa, respectively) with putative STK activity [58]. Moreover, the presence of gene BAE36_26360 coding for an 1843-aa long multi-sensor signal transduction multi-kinase, which contains several functional domains (i.e., histidine kinase domain, Ser/Thr kinase domain, ATP-binding domain, and Mg^2+^-binding domain), was also confirmed in the Rt24.2 genome (MAMO01000151) [58]. It is probable that the PssZ protein characterized in this study might function together with some of these STKs in phosphorylation/dephosphorylation reactions, influencing several physiological processes in rhizobial cells.

## 5. Conclusions

The *R. leguminosarum* bv. *trifolii* genome harbors the *pssZ* gene, which codes for a putative Ser/Thr phosphoprotein phosphatase. This gene is located in the large Pss-I polysaccharide synthesis cluster. Inactivation of *pssZ* affected several physiological and symbiotic properties of this bacterium, resulting in the loss of EPS synthesis, reduced growth kinetics and motility, altered surface properties, and disturbed symbiosis with clover. These observations confirm that the protein encoded by the *pssZ* gene is required for both EPS synthesis and proper functioning of *R. leguminosarum* bv. *trifolii* cells.

## Figures and Tables

**Table 1:** Bacterial strains, plasmids, and oligonucleotide primers used in this study.

Strains, Plasmids, and Primers	Characteristics	Source or Reference
***R. leguminosarum* bv. *trifolii***		
Rt24.2	Wild type, Rif^r^, Nx^r^	[38]
Rt297	Rt24.2 *pssZ::*mTn*5*SS*gusA*40, Sp^r^	This work
Rt297(pPL1)	Rt297 carrying *pssZ* on pBBR1MCS-2 vector, Km^r^	This work
Rt24.2(pPL1)	Rt24.2 carrying *pssZ* on pBBR1MCS-2 vector, Km^r^	This work
Rt24.2 (pBBR1MCS-2)	Rt24.2 carrying pBBR1MCS-2 vector, Km^r^	[39]
***E. coli***		
DH5α	*supE*44 Δ*lac*U169 (φ80 *lacZ*Δ M15) *hsdR*17 *recA*1*endA*1*gyrA*96 *thi*-1 *relA*1	[40]
S17-1	*thi pro hsdR^−^ hsdM^+^ recA* RP4-2-Tc::Mu-Km::Tn*7*	[41]
mTn5SS*gusA*40	miniTn*5* interposon containing a promoterless *gusA* gene, Sp^r^	[42]
**Plasmids**		
pBBR1MCS-2	*mob, lacZα*, cloning vector, Km^r^	[43]
pJBA21Tc	pMP220 containing *gusA*, Tc^r^	[44]
pPL1	pBBR1MCS-2 containing a 1.8-kb *Sal*I-*Xba*I fragment with the Rt24.2 *pssZ* gene**, Km^r^	This work
**Primers**	**Sequence (5′–3′) ^1^**	
gusF1	GCGTTACAAGAAAGCCGGGCAATT	This work
gusR1	GATCCAGACTGAATGCCCACAGGC	This work
gusR2	CAGCAATTGCCCGGCTTTCTTGTAA	This work
gusR3	GTCTGCCAGTTCAGTTCGTTGTTC	This work
Xba-Fw1	GGGTTTATCTAGACTGGCATCGGCAC	This work
Xba-Fw3	CAATCTCTATCTAGATGTGACCAACACC	This work
Xba-Fw4	GGACGCTCTAGATCTTTCAATCCTC	This work
Eco-Rw1	CCCGGTGAATTCGCCATCGTCAAC	This work
J44-Rw4	CAACCGCAGTTTCCACTTTGCACC	This work
J44-Rw5	GGATCTGAGATTCCTGATCAAGAAATG	This work
Sal-Rw2	CCTTCATATTGTCGACTCTGACCGTT	This work

Nx^r^, nalidixic acid resistance, Rif^r^, rifampicin resistance, Tc^r^, tetracycline resistance, Km^r^, kanamycin resistance, Sp^r^, spectinomycin resistance. ^1^ The sequences for the EcoRI, XbaI, and SalI restriction sites are underlined. *R. leguminosarum*: *Rhizobium leguminosarum*; *E. coli*: *Escherichia coli*.

**Table 2:** Sensitivity of the wild-type *R. leguminosarum* Rt24.2 and its derivatives to various stress factors.

Strain	Minimal Inhibitory Concentration ^a,b^		
	SDS (% *w*/*v*)	DOC (% *w*/*v*)	Ethanol (% *v*/*v*)
Rt24.2 (wt)	0.035 ± 0.005 ^B^	0.12 ± 0.05 ^B^	5.0 ± 0.25 ^A,B^
Rt297(*pssZ*)	0.025 ± 0.005 ^B,C^	0.11 ± 0.05 ^B^	4.25 ± 0.25 ^C^
Rt297(pPL1)	0.045 ± 0.005 ^A,B^	0.12 ± 0.05 ^B^	5.0 ± 0.25 ^A,B^
Rt24.2(pPL1)	0.050 ± 0.005 ^A^	0.14 ± 0.05 ^A^	5.5 ± 0.25 ^A^

^a^ The values are averages ± SD from three independent experiments with three biological replicates for each strain and treatment. ^b^ Data in the same column followed by different letters A, B, and C are significantly different (*p* < 0.05; one-way ANOVA). SDS: sodium dodecyl sulfate; DOC: sodium deoxycholate.

**Table 3:** Cell properties of the wild-type *R. leguminosarum* Rt24.2 and its derivatives determined using AFM analysis.

Property	Strain			
	Rt24.2	Rt297	Rt297(pPL1)	Rt24.2(pPL1)
Length (μm)	2.50 ± 0.21	2.83 ± 0.13	2.64 ± 0.22	2.58 ± 0.24
Width (μm)	0.78 ± 0.06	0.86 ± 0.05	0.80 ± 0.07	0.82 ± 0.05
Height (μm)	0.215 ± 0.027	0.184 ± 0.022	0.178 ± 0.015	0.204 ± 0.021
Roughness (nm)	2.786 ± 0.360	0.918 ± 0.240 *	2.903 ± 0.38	2.353 ± 0.26
DMT modulus (elasticity; GPa)	1.326 ± 0.187	2.501 ± 0.561 *	1.484 ± 0.206	1.524 ± 0.195
Adhesion (nN)	501.93 ± 49.74	257.00 ± 33.11 *	541.71 ± 36.00	609.43 ± 43.61 *

* Significantly different values between the wild-type Rt24.2 and the remaining strains (*p* < 0.05, Student’s *t*-test).
